# Canadian oncogenic human papillomavirus cervical infection prevalence: Systematic review and meta-analysis

**DOI:** 10.1186/1471-2334-11-235

**Published:** 2011-09-05

**Authors:** Andrea C Tricco, Carmen H Ng, Vladimir Gilca, Andrea Anonychuk, Ba' Pham, Shirra Berliner

**Affiliations:** 1 Li Ka Shing Knowledge Institute, St Michael's Hospital, (38 Shuter Street), Toronto, Ontario, (M5B 1T8), Canada; 2 School of Population and Public Health, University of British Columbia, (2206 East Mall), Vancouver, British Columbia, (V6T 1Z3), Canada; 3 Centre de Recherche du CHUL (CHUQ), l'Université Laval, (2705 boulevard Laurier), Québec, Québec, (G1V 4G2), Canada; 4 GlaxoSmithKline Biologicals, (Avenue Fleming 20), Wavre (1300), Belgium; 5 Health Policy Management and Evaluation, University of Toronto, (155 College Street), Toronto, Ontario, (M5T 3MT), Canada; 6 Toronto Health Economics and Technology Assessment, University of Toronto, (144 College Street), Toronto, Ontario, (M5S 3M2), Canada; 7 Department of Epidemiology, University of Western Ontario, (Kresge Building), London, Ontario, (N6A 5C1), Canada

**Keywords:** human papillomavirus, systematic review, meta-analysis, Bethesda system, cervical cancer

## Abstract

**Background:**

Oncogenic human papillomavirus (HPV) infection prevalence is required to determine optimal vaccination strategies. We systematically reviewed the prevalence of oncogenic cervical HPV infection among Canadian females prior to immunization.

**Methods:**

We included studies reporting DNA-confirmed oncogenic HPV prevalence estimates among Canadian females identified through searching electronic databases (e.g., MEDLINE) and public health websites. Two independent reviewers screened literature results, abstracted data and appraised study quality. Prevalence estimates were meta-analyzed among routine screening populations, HPV-positive, and by cytology/histology results.

**Results:**

Thirty studies plus 21 companion reports were included after screening 837 citations and 120 full-text articles. Many of the studies did not address non-response bias (74%) or use a representative sampling strategy (53%).

Age-specific prevalence was highest among females aged < 20 years and slowly declined with increasing age. Across all populations, the highest prevalence estimates from the meta-analyses were observed for HPV types 16 (routine screening populations, 8 studies: 8.6% [95% confidence interval 6.5-10.7%]; HPV-infected, 9 studies: 43.5% [28.7-58.2%]; confirmed cervical cancer, 3 studies: 48.8% [34.0-63.6%]) and 18 (routine screening populations, 8 studies: 3.3% [1.5-5.1%]; HPV-infected, 9 studies: 13.6% [6.1-21.1%], confirmed cervical cancer, 4 studies: 17.1% [6.4-27.9%].

**Conclusion:**

Our results support vaccinating females < 20 years of age, along with targeted vaccination of some groups (e.g., under-screened populations). The highest prevalence occurred among HPV types 16 and 18, contributing a combined cervical cancer prevalence of 65.9%. Further cancer protection is expected from cross-protection of non-vaccine HPV types. Poor study quality and heterogeneity suggests that high-quality studies are needed.

## Background

Human Papillomavirus (HPV) is one of the most prevalent sexually transmitted infections in the world [1]. Over 100 virus genotypes have been identified and at least 13 are classified as oncogenic or "high-risk" (HR) because they are known to cause cervical cancer or other genital cancers [2,3]. The association between HR HPV infection and cervical cancer has been well established in the literature with HR HPV DNA detected in nearly 100% of all cervical cancers [4-6].

For nearly half a century, cervical cancer prevention programs consisted mainly of cervical cancer screening for early detection of pre-cancerous lesions [7]. Although screening programs are effective, [8] cervical cancer remains a problem due to a large portion of women who remain unscreened or under-screened, [9-14] as well as false negative results on the Papanicolau test [15,16]. In 2009, an estimated 1,300 Canadian women developed cancer of the cervix and 390 died as a result of this disease [8]. The recent introduction of two prophylactic vaccines [17,18] offer further reduction of HPV, yet the most optimal primary and secondary prevention strategies remain unclear [19,20].

Baseline prevalence data is necessary to inform optimal prevention programs and evaluate current and future prevention strategies [21,22]. Since HPV is not a reportable disease in Canada, data on HPV prevalence are widely scattered in the literature and mainly reported among specific populations. We aimed to consolidate the prevalence of DNA-confirmed cervical HPV infection among Canadian females prior to immunization programs through a systematic review and meta-analysis.

## Methods

A systematic review protocol was compiled based on guidelines from The Cochrane Collaboration [23] and the Preferred Reporting Items for Systematic Reviews and Meta-Analyses [24]. The protocol is available upon request.

### Eligibility criteria and study selection

Studies reporting DNA-confirmed (i.e., through hybrid capture I or hybrid capture II (HCI or HCII) or polymerase chain reaction (PCR)) HPV prevalence estimates among Canadian females who were not previously vaccinated against HPV were included. Authors of studies not reporting the sample size were contacted and the study was excluded if the sample size could not be obtained. Inclusion was not limited by study design, publication status, year of dissemination or language of dissemination.

In order to ensure reliability, a training exercise was conducted prior to commencing the screening process. Two independent reviewers screened the search results for inclusion using a pre-defined relevance criteria form, obtained the full-text of potentially relevant articles and screened them to determine inclusion. Discrepancies were resolved by discussion or the involvement of a third reviewer.

### Information sources and search

Medical Subject Headings and text words related to the prevalence of HPV cervical infection among Canadian females were used to search MEDLINE (OVID interface, 1950 to October week 5, 2009), EMBASE (OVID interface, 1980 to 2009 week 44), The Cochrane Library (Wiley interface), and POPLINE (Knowledge for health interface 1970 to Nov 2, 2009). The electronic database search was supplemented by conducting a targeted search of Canadian public health websites (e.g., Public Health Agency of Canada, Health Canada, Institut National de Sante Publique du Quebec), websites of organizations that produce guidelines (e.g., Canadian Agency for Technologies in Health, Society of Obstetrics and Gynecologists of Canada), and vaccine manufacturer websites (e.g., GlaxoSmithKline, Merck). Furthermore, general Internet searches were conducted in Google and the first 60 unique hits were scanned. In addition, the reference lists of included studies were scanned, the authors' personal files were searched, and HPV experts in public health and industry were emailed to ensure that all potentially relevant data was obtained.

An experienced information specialist conducted all of the literature searches. The search strategy for the main (MEDLINE) search is presented in Additional File 1. It was peer reviewed by another information scientist using Peer Review of Electronic Search Strategies (PRESS) [25]. The main search was modified for the other databases, as necessary (full search strategies for the other databases available upon request).

### Data collection process and data items

A draft data abstraction form was developed, piloted, and modified as necessary. Two reviewers abstracted all of the data using the standardized data abstraction form, independently. Discrepancies were resolved by discussion or the involvement of a third reviewer.

The data abstracted included study characteristics, (e.g., study design, period of data collection, sample size, setting, province or territory and city of study conduct), participant characteristics (e.g., population, mean age and standard deviation), HPV detection method (e.g., HC-I or HC-II, PCR), PCR primers (e.g., MY09, MY11), number of HPV positive, as well as the overall HPV prevalence. As done in previous reviews of HPV prevalence, [26-28] we focused on HR HPV types commonly recognized to be associated with cervical cancer. These included the following HPV types: 16, 18, 31, 33, 35, 39, 45, 51, 52, 56, 58, 59, and 68 [3]. Specimens that were excluded from study analysis due to inadequate specimen integrity (e.g., negative for β-globin) were not included in the sample size.

Four of the included studies directly compared prevalence results for different tests (e.g., PCR versus HC-II) or methods (e.g., wood versus plastic spatula; cotton swab versus cytobrush) [29-32]. For these studies, prevalence results for the test or method found to be the most sensitive were abstracted. These included HC-II, [31,32] plastic spatula, [29] and cytobrush [30]. In some instances, multiple study publications reported data from the same population (i.e., companion reports). When this occurred, the report with the largest sample size was included and the other report(s) was used for supplementary data.

A commonly used and validated quality tool for observational studies does not exist [33]. As such, a generic methodological quality tool was developed and applied to all of the included studies, regardless of study design using a similar format that was used in a previous systematic review [34]. One item was added from The Cochrane Collaboration's Risk of Bias Tool on outcome reporting bias, which ensures that all outcomes that were assessed are reported in the study report [23]. The final tool consisted of the sampling strategy, sensitivity of sampling, timing of sampling, non-response bias, outcome reporting bias, and conflict of interest (Additional File 2).

### Age-specific prevalence synthesis

Studies reporting the age-specific prevalence of HR HPV infection were plotted and variation due to timing of data collection, HPV detection methods, and study location was noted. In order to ensure consistency across studies, only studies that collected samples in routine screening (i.e., routine Papanicolau test) were included in the plot. These results were described narratively and not combined via meta-analysis to view the wide heterogeneity observed across studies.

### Prevalence meta-analyses

HPV prevalence among routine screening populations was defined as the proportion of individuals who were positive for HR HPV infection divided by the total population tested for HPV infection. HPV type-specific prevalence among routine screening populations was defined as the proportion of females testing positive for the specific HR HPV type among all of those testing for HPV infection. These analyses only included studies of females engaged in routine screening.

HR HPV prevalence and HR HPV type-specific prevalence was also calculated for those testing positive for HPV infection. These analyses included studies of females engaged in routine screening, as well as those returning after a previous abnormal cytological test [35].

HPV type-specific prevalence by cytology or histology was defined as the proportion of individuals testing positive for the specific HR HPV type broken down by the cytological and/or histological category according to the 2001 Bethesda System (Additional File 3) [36]. Studies using routine screening, those returning after a previous abnormal cytological test, as well as those receiving a biopsy were included in this analysis. Individuals diagnosed with squamous cell carcinoma of the cervix and/or aden/adenosquamous carcinoma of the cervix were included in the confirmed cancer category.

Pooled estimates of type-specific prevalence of HR HPV infection were derived using a random-effects model [37]. The 95% CIs were derived based on a normal distribution. In order to produce conservative prevalence estimates and corresponding 95% CIs, studies reporting zero for the number of positives were imputed as being 0.5. Each HR HPV type was considered individually; hence prevalence estimates might include concomitant infection with other HPV types. All analyses were conducted in SAS 9.1 software (SAS Institute Inc., Cary, NC, USA).

## Results

### Literature search

**Figure 1:**
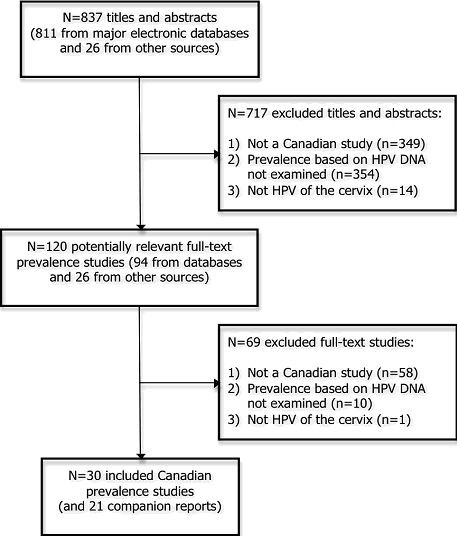
**Study flow**.

The literature search resulted in a total of 837 citations (i.e., titles and abstracts). Thirty study reports [29-32,35,38-62] plus 21 companion reports [27,28,63-81] fulfilled the inclusion criteria and were included after screening 120 full-text articles (Figure 1).

### Study characteristics

Data were collected from 1960 [42] to 2007 [48] (Table 1). Sample sizes varied from 46 [38] to 9,620 females [54]. Most of the included studies reported the actual number of HPV positive, except for one study, which reported weighted estimates [39]. Twenty-eight of the included studies were published in journals and two were theses [48,55]. Six studies reported infection with multiple HPV types (i.e., multiple infection), which was an average of 17.1% (median 4.4%) and ranged from 1.2% to 62.0% [31,39,40,49,58,62].

**Table 1:** Study characteristics

Reference	Study population; year of data collection; province(s)	Sample size	Mean age (SD) or age range	Method of detection	# cases‡	HPV types detected	Overall HPV prevalence (any type)†	HR HPV prevalence	Methodological quality components
Peng 1988 [30]	Patients with abnormal pap smears Dates NR Ontario	200	NR	In-situ hybridization	28 (HR16, 18)	Specific HPV types probed**§**	NA	14% (HR16, 18)	1.N, 2.Y, 3.U, 4.N, 5.Y, 6.N, 7.Y

Caussy 1990 [42]	Cases with cervical cancer, controls with CIN without cervical cancer 1960-1986 British Columbia	141	35.6 (24)	In-situ hybridization	31	HR-16, 18	22%	17.0%	1.N, 2.Y, 3.U, 4.Y, 5.N, 6.N, 7.Y

Duggan 1990 [29]	Patients referred to colposcopy clinic Dates NR Alberta	119	NR	Dot blot hybridization	28 (HPV 16/18/33)	HR-16, 18, 33 LR-6, 11	NA	23.5% (HPV 16/18/33)	1.N, 2.Y, 3.U, 4.N, 5.Y, 6.N, 7.U

Rohan 1991 [60]	University Students: Routine Screening 1990 Ontario	105	23 (median age)	PCR (E7/E6)	19	HR-16, 18, 33 LR-6, 11	12.3%	10.5%	1.Y, 2.Y, 3.Y, 4.N, 5.Y, 6.Y, 7.Y

Guijon 1993 [47]	Patients referred to colposcopy clinic 1985-1990 Manitoba	409	25.7 (NR)	In-situ hybridization	280	HR-16, 18, 33 LR-6, 11	NA	68.5% (HPV 16/18)	1.N, 2.Y, 3.Y, 4.N, 5.Y, 6.N, 7.Y

Brisson 1994 [41]	Patients referred to colposcopy clinic 1988-1989 Quebec	1140		Southern Blot	28.2 (6.6)	HR-16, 18 LR-6, 11	NA	24.3% (HPV-16)	1.N, 2.Y, 3.Y, 4.N, 5.Y, 6.N, 7.Y

Bosch 1995 [38]	Tumor biopsies of invasive cervical cancer 1989-1992 Province NR	46	NR	PCR (MY09/ MY11)	43	HR-16	93.5%	91.3%	1.NA, 2.Y, 3.NA, 4.N, 5.N, 6.Y, 7.Y

Duggan 1995 [43]* [27,68]	Cervical cancer tumour biopsies 1970-1990 Alberta	76	47.3 (16.7)	Dot blot supplemented by PCR	53	All	70.0%	65.8%	1.NA, 2.Y, 3.NA 4.Y, 5.N, 6.Y, 7.Y

Franco 1996 [46]	Cervical cancer tumour biopsies 1983-1990 Quebec	69	NR	PCR (GP5+/GP6+)	59	HR-16, 18, 31, 33, 35 LR-6, 11	85.5%	40.6% (HPV 16/18)	1.NA, 2.Y, 3.NA, 4.N, 5.N, 6.Y, 7.Y

Young 1997 [62]	General Public (Low-income, inner-city area): Routine Screening 1992-1995 Manitoba	1263	73% less than 30 years	PCR (MY09/ MY11)	411	HR-16, 18, 31 LR-6, 11	32.5%	18.0% (HPV 16/18)	1.N, 2.Y, 3.U, 4.N, 5.Y, 6.Y, 7.Y

Duggan 1997 [44]* [67,69,70]	Cervical biopsies NR Alberta	525	28.0	PCR	249	HR-16, 18, 31, 33, 35 LR-6, 11	47.4%	28.0% (HPV 16/18)	1.N, 2.Y, 3.U, 4.Y, 5.Y, 6.Y, 7. U

Michael 1999 [55]	University Students: Routine Screening NR Ontario	99	NR	PCR (MY09/ MY11)	40	HR-16, 18, 31, 33, 35 LR-6, 11	40.4%	10.1% (HPV 16/18)	1.N, 2.Y, 3.Y, 4.Y, 5.Y, 6.Y, 7.Y

Hankins 1999 [49]* [63,64,66,71]	HIV positive 1993-2000 Multiple provinces	375	32.5	PCR (MY09/ MY11)	252	HR-16, 18	67.2%	49.1%	1.N, 2.Y, 3.U, 4.Y, 5.Y, 6.Y, 7.Y

Richardson 2000 [58]	University Students: Routine Screening 1992-1993 Quebec	375	18-24	PCR (MY09/ MY11)	85	HR- [11] LR-6,11,53	22.7%	11.8%	1.N, 2.Y, 3.Y, 4.N, 5.Y, 6.Y, 7.U

Sellors 2000a [31]* [65,74,79]	General Public: Routine Screening 1998-1999 Ontario	955 (HC-II); 824 (PCR)	15-49	PCR (MY09/ MY11; HC-II	110	HR- [11] LR- [16]	13.3%	12.7% (HC-II)	1.Y, 2.Y, 3.Y, 4.Y, 5.Y, 6.Y, 7.Y

Sellors 2000b [35]	Patients recalled after an Abnormal Pap on Routine Screening 1996-1997 Ontario	200	31.5 (9.4)	HC-II/PCR (L1)	125	HR- [13] LR-6,11,42,53	62.5%	90.3% HSIL	1.N, 2.Y, 3.Y, 4.N, 5.Y, 6.Y, 7.Y

Ratnam 2000 [57]	General Public: Routine Screening 1996-1998 Newfoundland	2098	30 (NR)	HC-I/HC-II	227 (HR13)	HR- [13] LR-6,11,42,53	NA	10.8%	1.Y, 2.Y, 3.Y, 4.Y, 5.Y, 6.Y, 7.Y

Feoli-Fonseca 2001 [45]* [77]	Biopsies suspected of HPV Dates NR Quebec	691	26.1	PCR (MY09/ MY11/GP5+/GP6+)	484	HR- [13]	70.0%	92.0% (CIN III) 100% (carcinoma *in situ*)	1.NA, 2.Y, 3.NA, 4.Y, 5.N, 6.Y, 7.Y

Healey 2001 [50]* [72,73]	General Public (Inuit): Routine Screening 1999 Nunavut	1290	13-79	HC-II	333	HR- [13]		25.8%	1.Y, 2.Y, 3.Y, 4.Y, 5.Y, 6.Y, 7.Y

Tran-Thanh 2002 [61]* [80]	Cases with SIL or invasive cancer and controls with normal cytology and no history of cervical disease 1998-2000 Quebec	320	16-73	PCR (MY09/ MY11)	206	HR- [13]	64.4%		1.N, 2.Y, 3.Y, 4.N, 5.Y, 6.Y, 7.Y

Sellors 2002 [32]	General Public: Routine Screening 1999-2000 Ontario	156	50 and over	HCII/PCR	13	HR- [13] LR- [14]	NA	8.3% (HC-II)	1.N, 2.Y, 3.N, 4.N, 5.Y, 6.Y, 7.U

Richardson 2003 [59]* [81]	University Students: Routine Screening 1996-1998 Quebec	621	83% under age 27 years; mean age 23 (NR)	PCR (MY09/ MY11)	180	HR- [13]	29%	21.8%	1.N, 2.Y, 3.U, 4.N, 5.Y, 6.Y, 7.Y

Lytwyn 2003 [75]* [53]	Patients recalled after an Abnormal Pap: ASCUS or LSIL only 1995-1998 Ontario	105	30.3 (8.1)	HC-II	57 (HR13)	HR- [13] LR- [14]	NA	54.3%	1.Y, 2.Y, 3.Y, 4.Y, 5.Y, 6.Y, 7.Y

Koushik 2005 [52]* [76,78]	General Public: Cases with CIN from colposcopy clinics, controls with normal pap from screening. 2001-2003 Quebec	357 cases, 760 controls	32.0 (9.1) Cases, 31.7 (10.0) Controls	PCR (MY09/ MY11)	579	HR- [13]	51.8%	41.8%	1.N, 2.Y, 3.Y, 4.Y, 5.Y, 6.Y, 7.Y

Mayrand 2006 [54]	General Public: Routine Screening 2002-2004 Quebec, Newfoundland	9620	NR	HCII	591	HR- [13] LR- [24]	NA	6.1%	1.N, 2.Y, 3.Y, 4.Y, 5.Y, 6.Y, 7.Y

Ogilvie 2007 [56]	Sex trade workers or women with a history of alcohol or drug abuse 2004-2005 British Columbia	151	39.0	HC-II	43 (HC13)	HR- [13]	NA	28.5%	1.N, 2.Y, 3.Y, 4.N, 5.Y, 6.Y, 7.Y

Kapala 2007 [51]	General Public: Routine Screening Date NR Ontario	320	73% age 30 or greater	HC-II	92 (HR13)	HR- [13]	NA	28.8%	1.NA, 2.Y, 3.NA, 4.N, 5.Y, 6.Y, 7.U

Antionishyn 2008 [40]	Cervical biopsies 1995-1998 Saskatchewan	1355	NR	PCR (GP5+/GP6+)	753	HR- [13]	55.6%	56.8% CIN III (16 and 18)	1.NA, 2.Y, 3.NA, 4.N, 5.Y, 6.Y, 7.Y

Hamlin-Douglas 2008 [48]	General Public (Inuit): Routine Screening 2002-2007 Quebec	554	35.5 (14.4)	PCR (MY09/ MY11)	160	All	28.9%	20.4%	1.Y, 2.Y, 3.N, 4.Y, 5.Y, 6.Y, 7.Y

Moore 2009 [39]	Population-based estimates weighted by cytology 2004 British Columbia	4821	13-86	PCR (GP5+/GP6+)	810	All	16.8%	13.9%	1.NA, 2.Y, 3.NA, 4.Y, 5.Y, 6.Y, 7.N

Notes: * major publication and other references are companion reports, **‡ **number of women positive for human papillomavirus, † calculated by using the "number of cases" value as the numerator and the "sample size" as the denominator, expressed as a percent. **Abbreviations: **SD standard deviation, NR not reported, HC hybrid capture, PCR polymerase chain reaction.

Overall, 74% (17/23) did not address non-response bias, 53% (16/30) did not use a representative sampling strategy (i.e., they included individuals with risk factors for HPV and their results cannot be generalized to the general population), 17% (5/30) did not use adequate timing of sampling (e.g., retrospective instead of prospective), and 17% (5/30) did not use sensitive sampling techniques (e.g. hybridization). Seven studies out of 30 were not relevant to the non-response bias and response rate methodological quality items because they included data from cancer biopsies (Additional File 4) [38-40,43,45,46,51]. Excluding these studies, some of the others (35%, 8/23) did not report the response rate or used an inappropriate approach to calculate the response rate (9%, 2/23). Individual methodological quality components for each study are reported in Table 1.

### Age-specific HPV prevalence

**Figure 2:**
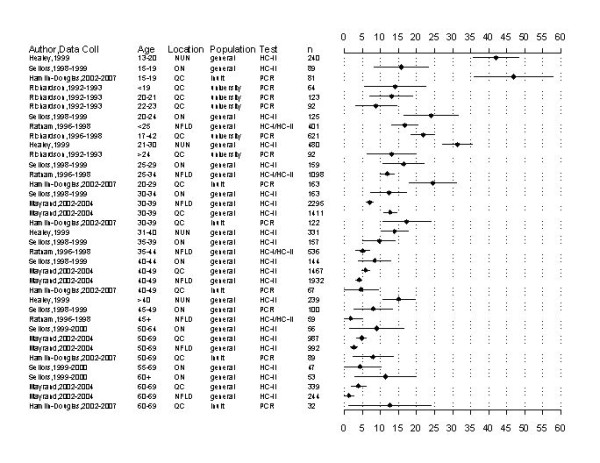
**Age-specific HPV prevalence**. **Abbreviations: **Coll collection, NUN Nunavit, HC hybrid capture, n total sample size, ON Ontario, QC Quebec, PCR polymerase chain reaction, NFLD Newfoundland.

The age-specific prevalence was generally highest among females less than 20 years of age and slowly declined as age increased, despite widespread heterogeneity across studies (Figure 2). HPV prevalence among females less than 20 years of age [31,48,50,58] ranged from 14.1% among university students [58] to 46.9% among Inuit females from Nunavut [48]. For women aged 20-25 years, HPV prevalence ranged from 13.0% among university students [58] to 24.0% among the general population of Ontario [31]. The prevalence among Nunavut women aged 21-30 years was 31.3% [50] and the prevalence of Inuit women from Quebec aged 20-29 years was 24.5% [48]. Ontario women aged 25-29 years had a prevalence of 16.4% [31] and Newfoundland women aged 25-34 years had a prevalence of 11.8% [57].

HPV prevalence among women aged 30-39 years [31,48,50,54,57] ranged from 7.0% in Newfoundland [54] to 17.2% among Inuit women from Quebec [48]. For women aged 40-49 years, [31,48,54] the prevalence ranged from 4.0% in Newfoundland [54] to 8.3% in Ontario [31]. Prevalence among women aged 50-59 years [32,48,54] ranged from 2.5% in Newfoundland [54] to 8.9% among women aged 50-54 years in Ontario [32]. For women aged 60-69 years, [48,54] the prevalence ranged from 1.2% in Newfoundland [54] to 12.5% among Inuit women from Quebec [48]. Finally, the prevalence among Ontario women aged greater than 60 was 11.3% [32]. Overall, age-specific HPV prevalence estimates were higher for other populations (i.e., those testing positive for HPV and HPV prevalence by histology/cytology) compared to the routinely screened populations (data not shown).

### Meta-analysis results

**Figure 3:**
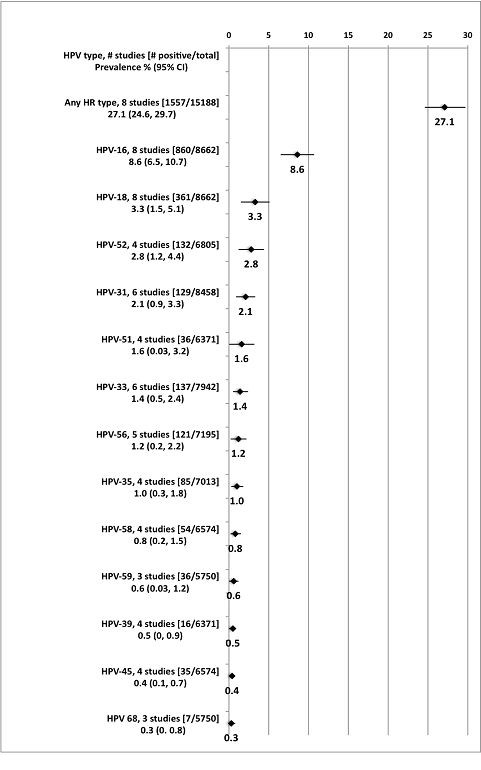
**HPV prevalence meta-analyses among all participants**. **Note: **Each point estimate and 95% confidence interval is a separate meta-analysis. **Abbreviations: **HR high risk, HPV human papillomavirus, CI confidence interval.

The meta-analysis results of HPV prevalence among routine screening populations are presented in Figure 3 and the three HPV types with the highest prevalence (HPV-16, -18, and -52) are highlighted here. The studies included in these analyses were conducted between 1990 and 2007, included an average of 1,019 individuals, and varied in their study location (British Columbia (BC), Manitoba (MB), ON, QC) [31,39,48,49,55,58-60,62]. For HPV-16, the pooled prevalence was 8.6% (95% CI: 6.5-10.7%); the lowest prevalence estimate was reported among university students (4.8%); [58] while the highest prevalence estimate was among participants from a low-income, inner city population (12.6%) [62]. The pooled prevalence for HPV-18 was 3.3% (95% CI: 1.5-5.1%) [31,39,48,49,55,58-60,62]. The lowest prevalence estimate was 0% among university students, [60] while the highest prevalence was 11.8% among a low-income, inner city population [62]. Four studies reported HPV prevalence for HPV-52 and their pooled prevalence was 2.8% (95% CI: 1.2-4.4%) [39,48,49,58]. The lowest prevalence estimate was 0% among a sample of university students, [58] while the highest prevalence estimate was 10.8% among women presenting to an sexually transmitted disease clinic [49].

**Figure 4:**
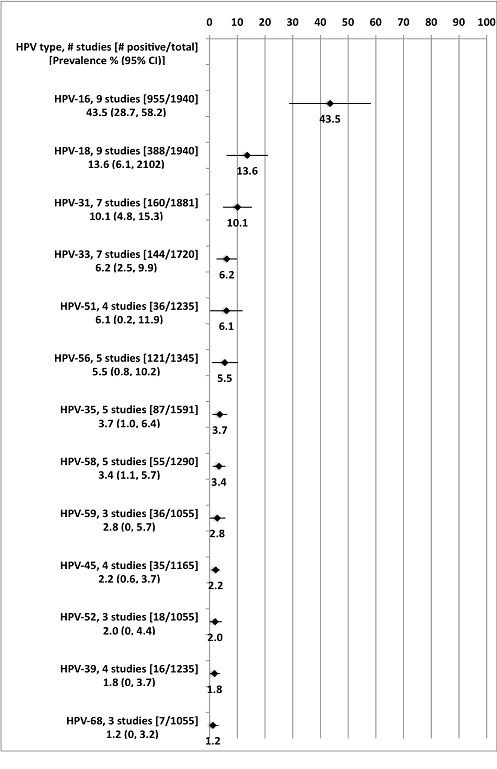
**HPV prevalence meta-analyses among HPV-infected participants**. Notes: Each point estimate and 95% confidence interval is a separate meta-analysis. One study (Aho 2003) included in the HPV prevalence among all participants was not included here because only one HPV type was examined (HPV-52). **Abbreviations: **HPV human papillomavirus, CI confidence interval.

The meta-analysis results for HPV prevalence among HPV-positive are presented in Figure 4 and the three HPV types with the highest prevalence (HPV-16, -18, and -31) are highlighted here. The studies included in these analyses were conducted between 1990 and 2007, included an average of 985 individuals, and varied in their study location (e.g., BC, MB, ON, QC) [31,35,39,48,55,58-60,62]. For HPV-16, the pooled prevalence from these nine studies was 43.5% (95% CI: 28.7-58.2%). The lowest prevalence was among Inuit women (19.4%) [48] and the highest prevalence was among participants with an abnormal Pap test (76.0%) [35]. For HPV-18, the pooled prevalence from these nine studies was 13.6% (95% CI: 6.1-21.1%). The lowest prevalence was among university students (0%), [60] while the highest prevalence was from a general population sample from a low-income, inner-city area (36.3%) [62]. For HPV-31, the pooled prevalence was 10.1% (95% CI: 4.8-15.3%), [35,39,49,58-60,62] and ranged from 2.3% among the general population [39] to 24.8% among participants with an abnormal Pap test [35].

The meta-analysis results according to the Bethesda classification system are presented in Table 2. The prevalence generally increased as the type of cervical lesion approached cervical cancer. The observed prevalence of combined HR HPV types for those with negative cytology or histology was 40.5% (95% CI: 26.4%-54.6%); 15.7% (95% CI: 9.3%-22.0%) for benign lesions; 40.5% (95% CI: 26.4%-54.6%) for ASC-US; 73.6% (95% CI: 55.3%-91.8%) for LSIL; and 89.9% (95% CI: 84.8%-95.1%) for HSIL.

**Table 2:** Prevalence of HPV by Bethesda classification system

HPV Type	Bethesda classification	# studies	Reference(s)	# cases	Total sample size	% Prevalence (95% CI) from meta-analysis
HR**‡**	Negative†	9	[31,35,39,48,49,51,52,54,57]	1451	15102	40.5% (26.4%-54.6%)

	Benign	2	[51]	42	275	15.7% (9.3%-22.0%)

	ASC-US	5	[31,39,48,51,53,54]	136	350	40.5% (26.4%-54.6%)

	LSIL	10	[31,35,39,45,48,49,51,52,54,57]	928	1563	73.6% (55.3%-91.8%)

	HSIL	8	[31,35,39,45,51,54,57,78]	559	628	89.9% (84.8%-95.1%)

	Cervical Cancer	2	[51,54]	2	3	*

16	Negative	6	[35,39-41,48,52]	473	6017	7.9% (4.9%-11.0%)

	Benign	1	[39]	2	250	*

	ASC-US	1	[48]	0	17	*

	LSIL	7	[35,39-41,45,48,52]	506	1925	28.3% (15.1%-41.5%)

	HSIL	7	[35,39-41,45,48,61]	516	1068	54.0% (45.6%-62.4%)

	Cervical Cancer	3	[38,43,61]	81	172	48.8% (34.0%-63.6%)

18	Negative	5	[35,39,40,42,48]	156	4891	3.6% (1.6%-5.6%)

	Benign	1	[39]	14	250	*

	ASC-US	1	[48]	2	17	*

	LSIL	6	[35,39,40,42,48,52]	81	1312	5.8% (3.6%-7.9%)

	HSIL	5	[35,39,40,42,48]	47	662	7.9% (3.8%-12.0%)

	Cervical Cancer	4	[38,42,43,80]	42	219	17.1% (6.4%-27.9%)

31	Negative	4	[35,39,40,48]	33	4849	2.1% (0.1%-4.1%)

	Benign	1	[39]	2	250	*

	ASC-US	1	[48]	0	17	*

	LSIL	5	[35,39,40,48,52]	76	1241	7.3% (2.7%-11.8%)

	HSIL	4	[35,39,40,48]	64	432	14.2% (4.0%-24.5%)

	Cervical Cancer	3	[38,43,61]	3	172	1.2% (0.0%-2.73%)

33	Negative	4	[35,39,40,48]	38	4849	0.6% (0.0%-1.3%)

	Benign	1	[39]	1	250	*

	ASC-US	1	[48]	2	17	*

	LSIL	4	[35,39,40,48]	35	884	3.6% (1.2%-6.0%)

	HSIL	4	[35,39,40,48]	24	432	5.6% (3.5%-7.8%)

	Cervical Cancer	3	[38,43,61]	4	172	2.1% (0.0%-4.2%)

35	Negative	4	[35,39,40,48]	28	4849	0.5% (0.3%-0.8%)

	Benign	1	[39]	1	250	*

	ASC-US	1	[48]	0	17	*

	LSIL	4	[35,39,40,48]	31	884	3.1% (0.3%-6.0%)

	HSIL	4	[35,39,40,48]	8	432	1.8% (0.5%-3.0%)

	Cervical Cancer	3	[38,43,61]	0	172	*

39	Negative	3	[39,40,48]	11	4770	0.8% (0.0%-2.0%)

	Benign	1	[39]	0	250	*

	ASC-US	1	[48]	1	17	*

	LSIL	3	[39,40,48]	6	860	0.6% (0.0%-1.5%)

	HSIL	3	[39,40,48]	2	374	*

	Cervical Cancer	2	[38,61]	1	96	*

45	Negative	3	[39,40,48]	31	4770	0.6% (0.4%-0.9%)

	Benign	1	[39]	0	250	*

	ASC-US	1	[48]	0	17	*

	LSIL	3	[39,40,48]	9	860	1.1% (0.4%-1.7%)

	HSIL	3	[39,40,48]	6	374	*

	Cervical Cancer	2	[38,61]	8	96	7.7% (2.4%-13.0%)

51	Negative	3	[39]	7	4470	*

	Benign	1	[39]	0	250	*

	ASC-US	1	[48]	1	17	*

	LSIL	3	[39,40,48]	2	860	*

	HSIL	3	[39,40,48]	3	374	*

	Cervical Cancer	2	[38,61]	0	96	*

52	Negative	4	[39,40,48,52]	41	5530	1.4% (0.1%-2.7%)

	Benign	1	[39]	0	250	*

	ASC-US	1	[48]	1	17	*

	LSIL	4	[39,40,48,52]	50	1217	2.9% (0.8%-5.1%)

	HSIL	3	[39,40,48]	9	374	2.4% (0.9%-4.0%)

	Cervical Cancer	2	[38,61]	3	96	2.6% (0.0%-7.1%)

56	Negative	3	[39,40,48]	74	4770	1.4% (0.8%-2.0%)

	Benign	1	[39]	5	250	*

	ASC-US	1	[48]	0	17	*

	LSIL	3	[39,40,48]	52	860	5.8% (3.2%-8.3%)

	HSIL	3	[39,40,48]	4	374	*

	Cervical Cancer	2	[38,61]	2	96	1.6% (0.0%-4.1%)

58	Negative	4	[35,39,40,48]	37	4849	1.0% (0.3%-1.6%)

	Benign	1	[39]	0	250	*

	ASC-US	1	[48]	0	17	*

	LSIL	4	[35,39,40,48]	22	884	2.3% (1.3%-3.3%)

	HSIL	4	[35,39,40,48]	11	432	2.0% (0.0%-4.3%)

	Cervical Cancer	2	[38,61]	0	96	*

59	Negative	3	[39,40,48]	25	4770	0.5% (0.0%-1.1%)

	Benign	1	[39]	2	250	*

	ASC-US	1	[48]	1	17	*

	LSIL	3	[39,40,48]	9	860	0.9% (0.3%-1.6%)

	HSIL	3	[39,40,48]	9	374	2.1% (0.7%-3.6%)

	Cervical Cancer	2	[38,61]	0	96	*

68	Negative	3	[39,40,48]	5	4470	*

	Benign	1	[39]	0	250	*

	ASC-US	1	[48]	0	17	*

	LSIL	3	[39,40,48]	1	860	*

	HSIL	3	[39,40,48]	2	374	*

	Cervical Cancer	2	[38,61]	1	96	*

Notes: *meta-analysis not feasible due to small numbers, †negative for intraepithelial lesion or malignancy, ‡ includes analyses of all HR types combined (i.e., multiple-infection). **Abbreviations: **atypical squamous cells of undetermined significance (ASCUS), low-grade squamous intraepithelial lesions (LSIL), HSIL high-grade squamous intraepithelial lesions (HSIL).

The most prevalent HPV type among those classified as negative for intraepithelial lesion or malignancy was HPV-16 (7.9%) and the prevalence for each of the other HR types was less than 5%. Among those classified with LSIL, the three most prevalent types were HPV-16 (28.3%), HPV-31 (7.3%), and HPV-18 (5.8%). Among those classified with HSIL, the three most prevalent types were HPV-16 (54.0%), HPV-31 (14.2%), and HPV-18 (7.9%). Among those with confirmed cervical cancer, the highest prevalence was observed for HPV-16 (48.8% [95% CI: 34.0-63.6%]) [38,43,61], followed by HPV-18 (17.1% (95% CI: 6.4-27.9%) [38,42,43,61], and HPV-45 (7.7%, [95% CI: 2.4-13.0%]) [38,61].

### Sensitivity analysis

We conducted a post hoc sensitivity analysis of the meta-analysis results for key study characteristics including study location, study design, HPV detection method, sample size, and methodological quality. Differences in prevalence estimates across the included studies were not identified (data not shown).

## Discussion

To our knowledge, this is the most comprehensive systematic review for a single country. Other HPV prevalence reviews included worldwide data and grouped all countries in North America together [27,28,65]. The past reviews included few Canadian studies, while data from 30 Canadian studies along with 21 companion reports were included here. Previous reviews focused on HPV prevalence among cervical cancer [27,28,65], HIV [82] or healthy individuals [83]. Our review includes data on the full spectrum of HR HPV infection, providing a more comprehensive understanding of the role of HPV genotypes in different manifestations of infection.

HPV prevalence data are required to provide information related to baseline HPV burden of disease when implementing vaccination programs. All of the included studies were either conducted prior to HPV vaccination or did not include vaccinated individuals. These data can be used to evaluate current HPV vaccination program in the future, including vaccine impact on HR genotype prevalence. The data are also useful for health economic evaluations and epidemiological modeling research. HPV is not a reportable disease so baseline prevalence data could only be obtained by synthesizing highly heterogeneous individual studies. A limited number of studies provide population-based estimates of HPV prevalence and only one provincial based study [39] was available for our meta-analyses.

The results can inform optimal vaccination program scheduling. Our findings indicate that HPV prevalence was highest among females aged less than 20 years. Some countries (such as Canada) have publicly funded vaccination programs targeting pre-adolescent and adolescent girls and our results support this approach. High age-specific HR HPV prevalence up to the age of 30 years stresses the need of evaluating the impact of catch-up vaccination programs in these older age groups.

Our results indicate that the prevalence of HR HPV varies among different subgroups of the population with indications of higher prevalence in those at greater risk of being under-screened. In our review, socially disadvantaged individuals (i.e., living in low-income, inner-city areas or Aboriginal communities) had the greatest HPV prevalence. Although declines in incidence and mortality due to routine screening have occurred, [84] false-negative Pap test results continue to raise important questions regarding optimal screening policies [15,16]. Our review identified a positive HPV test (e.g., 7.9% HPV-16 and 3.6% HPV-18) among females with a negative Pap test. Both tests have associated sensitivities and specificities, and while there is continuing research in identifying the best screening policies with these tests, vaccination remains an effective method to prevent infection with the major HR HPV types [85]. In addition to errors due to diagnostic sensitivity and specificity, 12% of Canadians never go for a Pap test [9]. Individuals less likely to obtain the Pap test include those of lower education, [10,12,13] lower income, [10,12,13] recent immigrants, [14,86] ethnic minorities, [11] living in rural areas, [11] and those who are obese [87]. These groups of individuals merit special attention when implementing and evaluating HPV vaccination programs.

The HPV vaccines protect against oncogenic strains HPV-16 and -18. These two HPV types contributed a combined prevalence of 12% for HR HPV infection and 66% of cervical cancer, which potentially can be prevented by the vaccines assuming 100% efficacy and long-lasting immunity. These estimates are slightly lower than previous reviews that reported this estimate as being approximately 70%, [26] indicating that our results might be conservative. Emerging data showing cross-protection against several non-vaccine HR HPV types is encouraging. According to the bivalent vaccine product monograph, protection against HPV-31 (according to protocol cohort) and -45 (total vaccinated cohort) was also observed [17]. These two HR HPV types contributed an estimated combined prevalence of 8.9% (1.2% for HPV-31 and 7.7% for HPV-45) for cervical cancer, indicating that additional cancer protection is a possibility. According to the quadrivalent vaccine product monograph, there was some cross-protection against HPV-31, but not against -45 [18]. Ongoing follow-up of clinical trials and national registries can help understand the potential impact of cross-protection on HPV-related cancer reduction.

Our results are similar to previous reviews of HPV prevalence. The overall HR HPV prevalence observed in our systematic review was 27.1% among screening populations, which is lower than the prevalence for any HPV infection in HIV positive women (36.3%) reported in a previous review, as expected [82]. In addition, another previous review found that HPV prevalence peaked in younger women (≤ 25 years of age) and decreased with age, which is consistent with our results [83]. Similar to another review, [65] we found that there was an inconsistent relationship between HPV prevalence in HSIL versus cervical cancer cases across different HPV types. For example, we found a higher HPV prevalence in HSIL versus cervical cancer (including squamous cell carcinoma of the cervix and/or aden/adenosquamous carcinoma of the cervix) for HPV 16 (54.0% HSIL versus 48.8% cervical cancer), 31 (14.2% HSIL versus 1.2% cervical cancer), and 33 (5.6% HSIL versus 2.1% cervical cancer), whereas the reverse was true for HPV 18 (7.9% HSIL versus 17.1% cervical cancer) and 52 (2.4% HSIL versus 2.6% cervical cancer). The relationship between HSIL and cervical cancer could not be explored for the other HPV types, due to insufficient data. Furthermore, many of the cervical cancer estimates were based on a small number of studies, making these results difficult to interpret.

We found that multiple infections occurred in 17.1% of the included prevalence estimates. Multiple infections occurred in 11.2% of the prevalence estimates in a review of invasive cervical cancer [26] and 11.9% in a review limited to women infected with HIV [82]. Factors that can explain the difference in multiple infections across reviews include different duration of specific types of infection (i.e., natural clearance of one or several types but persistence of the main one); and/or potentially different number of new sexual partners during the last months in females with LSIL, HSIL and those with cervical cancer or HIV.

Compared to different regions throughout the world, HPV prevalence in Canada is moderate. One review found that the highest prevalence of HPV among middle-aged women (35-50 years) was observed for Africa, Central and South America, and the United States (approximately 20%), while a lower prevalence was observed in Asia, Australia, Europe, Middle East, and Canada (approximately 15%) [83]. Furthermore, the proportion of cancer cases associated with HPV 16 and 18 was found to be the highest in Africa (94.2%), moderate in North America (89.2%), and the lowest in Asia (68%) [26].

Limitations of this systematic review include that the meta-analyses were based on each HPV type individually so may include concomitant infection. Data were also pooled across studies using different tests to measure the presence of HPV (e.g., hybridization, PCR, HC) and these tests vary in their sensitivity and specificity. For example, HC-II has been found to be a more sensitive test versus HC-I [88]. The methodological quality of the included studies was variable; less than half used a representative sampling strategy and many had small sample sizes.

Population-based, high-quality, HPV prevalence studies are warranted. These studies should report age-specific prevalence, overlap between single and multiple infection, and HPV type-specific infection. A recently published prevalence study from the United Kingdom is an excellent example of such a study [89]. These data can lead to a better understanding of HPV prevalence, as well as the impact of new prevention programs at the national and international levels.

## Conclusions

Prevalence data of cervical human papillomavirus infection is necessary to inform optimal prevention programs and evaluate current prevention strategies. Previous HPV prevalence reviews included worldwide studies and focused on HPV prevalence among cervical cancer, HIV or healthy individuals. To our knowledge, this is the most comprehensive systematic review for a single country and the data can be used in health economic evaluations and epidemiological modeling research to help inform public health policy. Our results show that the highest prevalence occurred among females < 20 years of age, yet prevalence remained high among women up to 30 years of age.

## Competing interests

ACT, CHN, and SC have been paid consultants for vaccine-related products from GlaxoSmithKline, Canada. VG has received consultancy fees from GlaxoSmithKline and Merck Frosst Canada. AA is an employee of GlaxoSmithKline Biologicals. The funding agreement ensured the authors' independence in designing the study, interpreting the data, writing, and publishing the report.

## Authors' contributions

ACT designed and coordinated the study, participated in data collection and analysis, and drafted the manuscript. CHN participated in data collection and analysis and helped to draft the manuscript. VG helped to draft the manuscript. AA helped to draft the manuscript. BP conceived of the study, participated the study design and coordination, and helped to draft the manuscript. SB participated in data collection and analysis and helped to draft the manuscript. All authors read and approved the final manuscript.

## Pre-publication history

The pre-publication history for this paper can be accessed here:

http://www.biomedcentral.com/1471-2334/11/235/prepub

## Supplementary Material

Additional file 1 **Search strategy for MEDLINE**. Search strategy used in systematic review. Click here for file

Additional file 2 **Methodological quality and risk of bias tool**. Tool used to assess methodological quality of studies included in systematic review. Click here for file

Additional file 3 **Bethesda classification system**. Classification system for cervical cancer used in analysis of results. Click here for file

Additional file 4 **Methodological quality of the included studies**. Results of the methodological quality tool. Click here for file
