# Supplementary material for: Canadian oncogenic human papillomavirus cervical infection prevalence: Systematic review and meta-analysis
Source: BMC Infect Dis. 2011 Sep 5;11:235. doi: 10.1186/1471-2334-11-235 (PMC3185279; doi:10.1186/1471-2334-11-235)
Supplement: Additional file 1 — Search strategy for MEDLINE. Search strategy used in systematic review. [file 1471-2334-11-235-S1.DOC]

**Appendix 1: Search strategy for MEDLINE**

Ovid MEDLINE(R) 1950 to October Week 3 2009

| **#** | **Searches** |
| --- | --- |
| 1 | Uterine Cervical Neoplasms/ |
| 2 | Cervical Intraepithelial Neoplasia/ |
| 3 | exp Papillomavirus Infections/ |
| 4 | exp Papillomaviridae/ |
| 5 | Human papillomavirus.ti. |
| 6 | human papillomavirus.tw. |
| 7 | HPV.tw. |
| 8 | or/1-7 |
| 9 | exp canada/ |
| 10 | (canada or ontario or quebec or british columbia or alberta or manitoba or saskatchewan or newfoundland or new brunswick or nova scotia or prince edward island or yukon or nunavut).tw. |
| 11 | 9 or 10 |
| 12 | risk*.tw. |
| 13 | risk/ |
| 14 | cohort studies/ |
| 15 | group*.tw. |
| 16 | (cohort and stud*).tw. |
| 17 | prevalence/ |
| 18 | prevalence$.tw. |
| 19 | prevalence studies/ |
| 20 | or/12-19 |
| 21 | 8 and 20 |
| 22 | 21 and 11 |
