# Supplementary material for: Canadian oncogenic human papillomavirus cervical infection prevalence: Systematic review and meta-analysis
Source: BMC Infect Dis. 2011 Sep 5;11:235. doi: 10.1186/1471-2334-11-235 (PMC3185279; doi:10.1186/1471-2334-11-235)
Supplement: Additional file 2 — Methodological quality and risk of bias tool. Tool used to assess methodological quality of studies included in systematic review. [file 1471-2334-11-235-S2.DOC]

**Appendix 2: Methodological quality and risk of bias tool**

| 1. Describe efforts to address non-response bias | Score “Yes” if efforts to address nonresponse bias (through comparing responders to nonresponders) were reported. Score “No” if these items not addressed in the paper. Score “Unclear” if it is uncertain whether these were addressed. This item should be scored as not applicable if the study only used data from cancer biopsy samples. |
| --- | --- |
| 2. Study is free from outcome reporting bias | Score “Yes” if there is no evidence that outcomes were selectively reported (e.g. all relevant outcomes in the methods section are reported in the results section). Score “No” if some important outcomes are subsequently omitted from the results. Score “unclear” if not specified in the paper. |
| 3. Response rate reported | Score “Yes” if the response rate was reported as the # of participants in the study divided by # of people invited to take part. Score “No” if another calculation was used to derive the response rate. Score “Unclear” if the calculation for the response rate was not reported in the paper. This item should be scored as not applicable if the study only used data from cancer biopsy samples. |
| 4. Representative sampling strategy | Score “Yes” if the investigators attempted to achieve a sample of participants that represents the larger population from which they were drawn. Score “No” if an unpresentative sample was used (e.g., a single-centre sample). Score “Unclear” if this information is not reported in the paper. |
| 5. Adequate timing of sampling | Score “Yes” if the investigators used prospective sampling. Score “No” if the investigators used retrospective sampling. Score “unclear” if this information is not reported in the paper. |
| 6. Use of a sensitive sample | Score “Yes” if the investigators used hybrid capture or polymerase chain reaction. Score “No” if the investigators used older sampling methods (e.g., southern blot). Score “Unclear” if this information is not reported in the paper. |
| 7. Study is free from conflict(s) of interest | Score “Yes” if conflict(s) of interest reported and a public funding organization provided support for the study (e.g., government, university). Score “Unclear” if the conflict of interest or funding source was not reported. Score “No” if conflict of interest reported (e.g., private industry funding). |
