# Supplementary material for: Canadian oncogenic human papillomavirus cervical infection prevalence: Systematic review and meta-analysis
Source: BMC Infect Dis. 2011 Sep 5;11:235. doi: 10.1186/1471-2334-11-235 (PMC3185279; doi:10.1186/1471-2334-11-235)
Supplement: Additional file 3 — Bethesda classification system. Classification system for cervical cancer used in analysis of results. [file 1471-2334-11-235-S3.DOC]

**Appendix 3: Bethesda classification system**

The Bethesda classification system classifies cervical cytology as being 1) negative for intraepithelial lesion or malignancy, 2) having epithelial cell abnormalities or 3) other. Epithelial cell abnormalities can be classified as squamous cell or glandular cell abnormalities. These can be further classified by the severity/extent of the abnormality as follows:

1) Squamous cell abnormalities, including

a) Atypical squamous cells (ASC) of undetermined significance (ASC-US) or cannot exclude high-grade squamous intraepithelial lesions (HSIL; ASC-H)

b) Low-grade squamous intraepithelial lesions (LSIL), including HPV/mild dysplasia/cervical intraepithelial neoplasia (CIN) 1

c) HSIL, including moderate and severe dysplasia, carcinoma in situ; CIN 2 and CIN 3

d) Squamous cell carcinoma

2) Glandular cell abnormalities, including

a) Atypical glandular cells (AGC)

b) Atypical glandular cells favoring neoplastic

c) Endocervical adenocarcinoma in situ (AIS)

d) Adenocarcinoma
