# Supplementary figures and images for: Canadian oncogenic human papillomavirus cervical infection prevalence: Systematic review and meta-analysis
Source: BMC Infect Dis. 2011 Sep 5;11:235. doi: 10.1186/1471-2334-11-235 (PMC3185279; doi:10.1186/1471-2334-11-235)

**Appendix 4: Methodological quality of the included studies (% of studies scoring Yes, Unclear or No)**


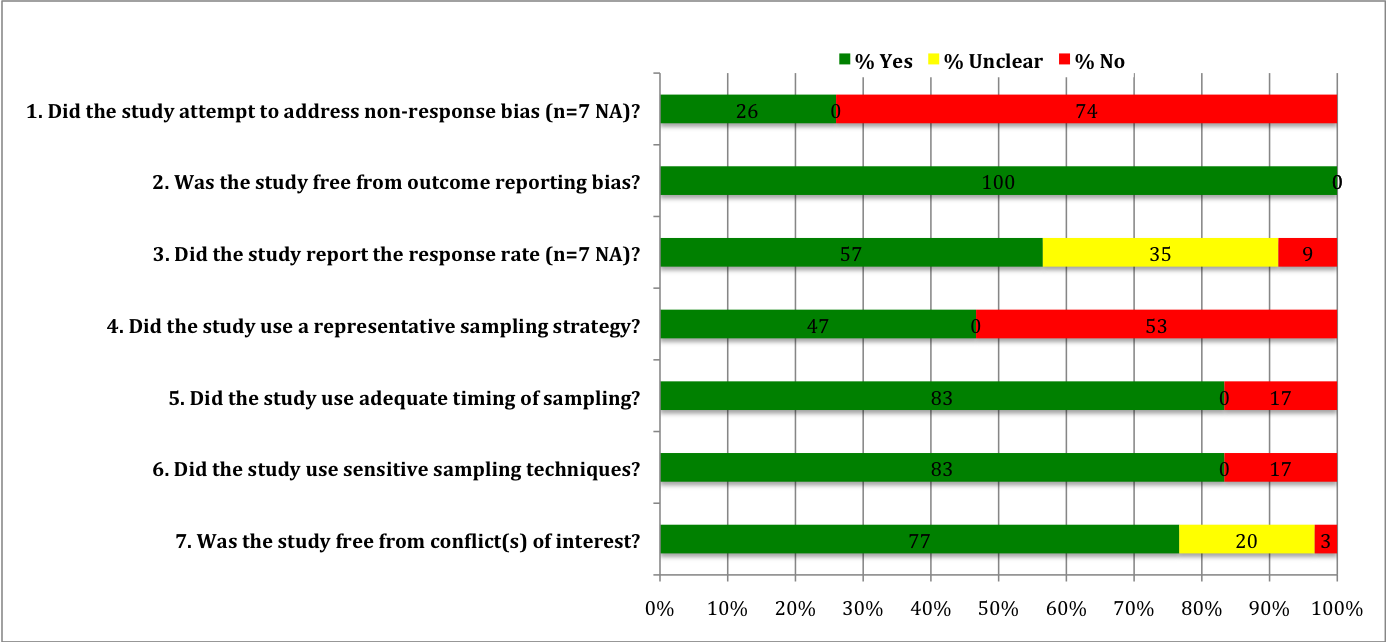

Supplement: Additional file 4 — Methodological quality of the included studies. Results of the methodological quality tool. [file 1471-2334-11-235-S4.DOC]
